# Complexity of Viral Epitope Surfaces as Evasive Targets for Vaccines and Therapeutic Antibodies

**DOI:** 10.3389/fimmu.2022.904609

**Published:** 2022-06-17

**Authors:** Nathaniel L. Miller, Rahul Raman, Thomas Clark, Ram Sasisekharan

**Affiliations:** ^1^Harvard Massachusetts Institute of Technology (MIT) Division of Health Sciences and Technology, Massachusetts Institute of Technology, Cambridge, MA, United States; ^2^Koch Institute for Integrative Cancer Research, Massachusetts Institute of Technology, Cambridge, MA, United States; ^3^Department of Biological Engineering, Massachusetts Institute of Technology, Cambridge, MA, United States

**Keywords:** epitope, paratope, glycoepitope, antibody, escape, SARS-CoV-2, N-glycan, repurposing

## Abstract

The dynamic interplay between virus and host plays out across many interacting surfaces as virus and host evolve continually in response to one another. In particular, epitope-paratope interactions (EPIs) between viral antigen and host antibodies drive much of this evolutionary race. In this review, we describe a series of recent studies examining aspects of epitope complexity that go beyond two interacting protein surfaces as EPIs are typically understood. To structure our discussion, we present a framework for understanding epitope complexity as a spectrum along a series of axes, focusing primarily on 1) epitope biochemical complexity (e.g., epitopes involving N-glycans) and 2) antigen conformational/dynamic complexity (e.g., epitopes with differential properties depending on antigen state or fold-axis). We highlight additional epitope complexity factors including epitope tertiary/quaternary structure, which contribute to epistatic relationships between epitope residues within- or adjacent-to a given epitope, as well as epitope overlap resulting from polyclonal antibody responses, which is relevant when assessing antigenic pressure against a given epitope. Finally, we discuss how these different forms of epitope complexity can limit EPI analyses and therapeutic antibody development, as well as recent efforts to overcome these limitations.

## 1 Introduction

Viral surface proteins are typically the immunodominant antigens that are targeted for antibody-mediated neutralization by the humoral immune response by the host. These viral proteins present numerous surfaces known as epitopes which are recognized by antibodies that are generated by the host immune system to specifically bind to these virus epitopes *via* the antibody’s functional ‘paratope’ domain in an epitope-paratope interaction (EPI). EPIs are key aspects of the dynamic interplay between the virus and the host immune response to neutralize the virus.

Host antibody responses upon viral infection vary widely depending on the virus and the host’s exposure history to the virus, homologous viruses, and vaccines. Hosts that have been previously infected or vaccinated typically possess neutralizing antibodies (nAbs) against vulnerable virus epitopes which protect the host from infection upon viral exposure, with the nAb titer often correlating with the degree of protection against future infections (1, 2). However, for certain viruses pre-existing antibodies resulting from infections of different sub-types may recognize but not effectively neutralize the virus, which may result in paradoxically worse disease in a mechanism known as antibody-dependent enhancement (ADE) (3–5). ADE may occur for viruses such as Dengue (DENV) that are capable of infecting cells possessing antibody Fc receptors (e.g., monocytes and macrophages), wherein non-neutralizing antibodies binding to DENV surface proteins enhance affinity and infectivity of DENV virions for these cell types, which facilitates infection and exacerbates disease course (6, 7).

Beyond viruses that do not infect *via* Fc-mediated mechanisms, poorly-neutralizing antibodies are undesirable as they may not protect the host from future exposures and thus lead to reinfection, though non-neutralizing antibodies can still play key roles in protection *via* Fc function (8–10). This neutralization ‘escape’ dynamic occurs, for example, in the case of influenza A strains and SARS-CoV-2 variants featuring mutations in vulnerable epitopes resulting in reinfection of hosts whose antibodies developed during prior infection or vaccination no longer effectively recognize the mutated epitopes (11–13). Antibody escape may be more or less pronounced depending on the host’s exposure history, with certain viruses tending to leave an imprint on the host antibody response based on the host’s first exposure to the virus in a mechanism known as original antigenic sin/seniority (14–16), which can occur divergently for antibodies (Abs) generated *via* vaccination versus infection as in the case of SARS-CoV-2 mRNA vaccines (17).

In this way, viruses experience continued pressure to evolve mutations in vulnerable epitopes toward acquiring the ability to escape existing antibodies and reinfect hosts. Likewise, hosts continually evolve new (in response to reinfection or additional vaccination) or matured (resulting from accumulation of somatic mutations within memory B cells) antibodies to neutralize viruses bearing mutated or homologous epitopes (18–20), wherein these responses are modulated by antigenic exposure history (21). As this continual evolutionary process that drives much of annual viral morbidity occurs as a result of EPI dynamics, studying EPIs enables us to improves our understanding of antigenic pressure-driven viral evolution (22, 23) that typically occurs in immunodominant epitopes (24, 25). Further, two of humanity’s best tools to alleviate viral disease burden are vaccines and therapeutic monoclonal antibodies (mAbs) which function directly or indirectly *via* EPIs to enhance protection against and resolution of viral infections. Of particular interest, certain individuals evolve broadly neutralizing antibodies (bnAbs) that retain functionality across many variants of the same virus as well as cross-neutralize evolutionarily-related viruses (26–30). Therefore, enhanced understanding of EPIs can translate to design of more effective vaccines and therapeutics that reduce the global burden of viral diseases (31–36). Likewise, improved modeling of EPIs and escape interactions may serve as the mechanistic basis for rapidly identifying antigenic drift on newly observed strains/variants toward guiding public health responses.

A variety of experimental approaches and computational tools have been developed to map virus antigenic landscapes, which have become increasingly valuable during the global response to address SARS-CoV-2 and subsequent waves of variants of concern. Experimental approaches include deep mutational scanning of immunodominant domains (11, 37–40), characterization of synthetic antigens bearing combinations of common mutations (41), live virus escape studies of known variants (41–43), longitudinal analyses of antigenic evolution during chronic infections (44–46), and pseudoviral antigenic evolution under therapeutic antibody pressure (28, 47–50). Computational approaches include prediction of escape hotspots and antigenic relationships between these hotspots based on structural complexes (51–54), molecular dynamics simulations (55–59), and calculators derived from deep mutational scanning datasets (60). These studies have provided detailed molecular, structural, and computational descriptions of the roles of specific mutations and combinations of mutations in escaping binding and neutralization by therapeutic monoclonal antibodies and vaccine/infection-induced polyclonal antibodies. Importantly, these studies have also highlighted the complexity of the epitope surface going beyond the direct protein-protein interactions between epitope and paratope.

Epitopes on pathogenic surface proteins have varying levels of conformational, dynamic, and post-translational complexity, all of which can have significant effects on the binding, specificity and neutralization potential of an antibody targeting that epitope. Conformationally, epitopes range from relatively simple, in that they are comprised of a linear stretch of amino acids on a monomeric protein domain, to complex, in that they are discontinuous and sample distinct conformations in a monomeric or even multimeric (quaternary) assembly of protein domains on the viral surface. Additionally, epitopes on viral surface proteins are not static in time. Many undergo significant conformational changes, including rearrangement of entire protein domains across the various stages of the viral life cycle. Finally, post-translational modifications such as glycosylation of viral surface proteins can either mask the underlying protein epitope surface or be part of epitope surfaces that are targeted by host antibodies as epitope constituents.

Glycans as epitope constituents have been most widely appreciated and interrogated through the study of HIV glycoprotein 120 (gp120), wherein researchers have sought to specifically induce and characterize the development of anti-glycan Abs (61, 62). Such complex glycoepitopes occur in a variety of formats, including glycan-protein epitopes (GPEs) in which the antibody paratope simultaneously engages both protein and glycan, and topological glycoepitopes in which the antibody recognizes an exclusively glycan-based epitope without *direct* dependence on the underlying protein surface (63–67). A variety of antibodies targeting GPE epitopes have been isolated against HIV gp120, which include the PG- and PGT- series that interact directly with gp120 N-glycans alongside their protein epitopes (26, 68–70), the VRC01-class antibodies whose maturation is shaped by epitope-adjacent glycans and can also involve direct interaction with the N276 glycan (71–73), and the extremely-potent VRC26-CAP256 lineage which displays a remarkable spectrum of sensitivity to N160 glycan deletion that ranges from complete escape for VRC26.01 to enhanced neutralization potency for VRC26.09 (74–76). 2G12 is the prototypic anti-glycan topological antibody, yet the class is potentially more broadly occurring with recent discovery of Fab-dimerized glycan-reactive (FDG) Abs (77) that similarly recognize high-mannose N-glycans in clustered topologies.

Beyond inclusion of glycans, epitope complexity can result from epitope secondary, tertiary, and quaternary structure which can present an epitope in multiple formats depending on viral capsid arrangement (3, 31, 78–80). Further, viral surface protein dynamics can drive epitope complexity, as epitopes on dynamic surfaces may present in diverse conformational states that affect antibody accessibility as observed for certain SARS-CoV-2 receptor binding domain (RBD). RBD epitopes are exposed variably across spike conformational states, wherein relative state occupancy varies across variants and state occupancy shifts can plausibly result in escape from antibodies targeting epitopes only accessible in “open” states (81–83). Therefore, it is important to analyze EPIs in the context of epitope complexity that extends beyond sequence conservation, which is often used to assess similarity of epitope surfaces across viral strains through evolution and antigenic drift. In this regard, it is also important to consider epitope complexity resulting from the three-dimensional protein structure of antigens, as mutation of sites outside of or adjacent to epitope residues directly contacting the antibody paratope or framework can allosterically modulate the physiochemical properties of epitope surfaces (84–89). These indirect network effects are critical to consider especially for variants featuring large numbers of mutations within the same epitope network as occurs in the SARS-COV-2 Omicron variant (11, 13, 49, 90), and inter-residue interaction networks are particularly well-suited for modeling these indirect interactions (51, 52, 91).

In this review, we describe distinct epitope features driving epitope complexity, including biochemical complexity resulting from factors such as glycosylation, conformational complexity resulting from antigen quaternary structural dynamics, and epistatic and allosteric complexity resulting from epitope secondary and tertiary structure. We include specific examples of each form of epitope complexity, and describe recent publications on efforts to model, analyze, and engineer epitope-paratope interactions that accommodate epitope complexity. Specifically, we present examples of network and structure-based analyses of complex viral epitope surfaces from the standpoint of improving design and repurposing of therapeutic antibodies and mapping antigenic drift, as performed by us and others. These approaches include functional characterization of a cross-neutralizing Henipavirus antibody (92), developing a framework for antibody-glycan interactions (63), modeling N-glycan topology toward repurposing the antibody 2G12 (93), mapping antigenic orthogonality between and within overlapping RBD epitopes (51), and rapidly mapping the mutational landscape of the Omicron sub-variants (52, 81).

## 2 Complexity of Epitope Surfaces on Viral Pathogens: Challenges and Opportunities

**Figure 1:**
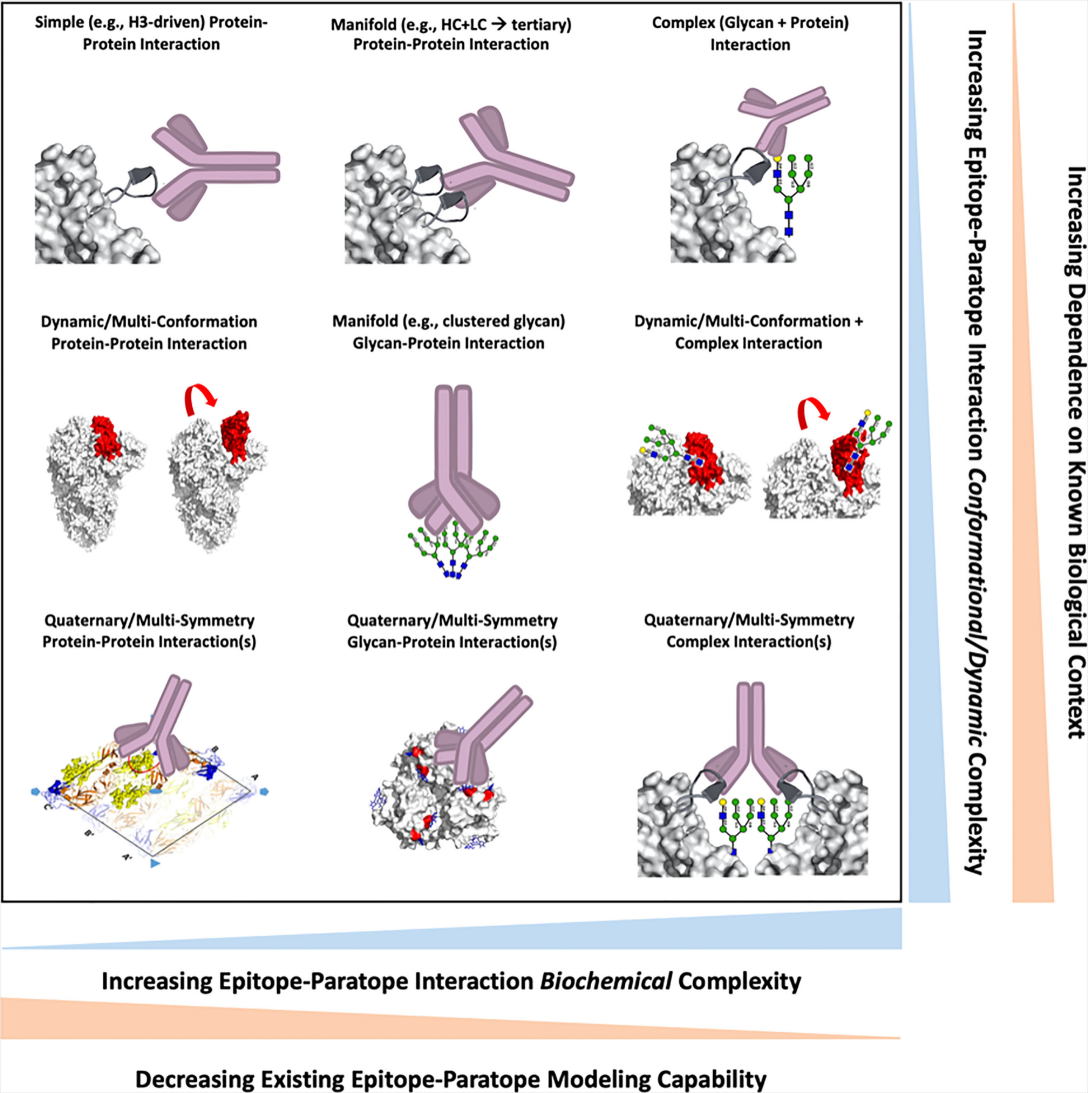
A survey of epitope-paratope interaction formats between antibodies and viral epitopes are shown, wherein each interaction is positioned along two axes describing interaction *biochemical* (x-axis) or *conformational/dynamic* (y-axis) complexity. Increasingly biochemically-complex interactions are inversely correlated with existing structural and computational modelling capabilities. Increasingly conformationally- or dynamically-complex epitopes require an increasing amount of existing structure-function knowledge of the viral antigen for epitope analyses and antibody design.

The range of epitope complexity presented on viral surface proteins drives ease of characterizing epitope-paratope interaction (EPI), availability of standard methods and tools to analyze the EPI and engineer antibodies against the epitope, and the amount of existing biological information/context required for such endeavors. To frame our perspective on epitope complexity, we first illustrate epitope complexity along two axes: 1) biochemical complexity involving factors such as glycosylation, and 2) conformational complexity involving epitope secondary, tertiary, and quaternary structure as well as epitope dynamics (**Figure 1**). We further annotate each axis with tradeoffs in EPI analysis and Ab engineering that come with increasing epitope complexity, and also plot a set of example epitope-paratope interaction formats within this space. With this framework in mind, we describe a set of EPI investigations and applications by ourselves and others for viral epitopes of varying complexity.

### 2.1 Antibody Cross-Reactivity in the Context of Epitope Complexity

#### 2.1.1 Cross-Reactivity of Abs Targeting Monomeric Protein Epitopes

In a straightforward use-case that the reader is likely familiar with, antibodies targeting monomeric protein epitopes can be repurposed and used as engineering templates to target homologous epitopes on evolutionarily-related viruses with high success rates. Antibodies targeting protein epitopes are widely available as they tend to be dominant in immune repertoires and exhibit high-affinity, prompting a selection bias in Ab isolation workflows and subsequent experimental or structural characterization. Further, computational tools [e.g., Rosetta (94–96)] and databases [e.g., SAbDab (97)] facilitate analysis of EPIs as these programs have been primarily designed with protein-protein EPIs in mind. Epitopes for repurposing can be rapidly identified according to epitope sequence conservation using basic multiple sequence alignment algorithms. In particular, study of flaviviruses has yielded extensive details on cross-neutralization of related viruses by antibodies targeting protein epitopes (98–100), though this has also been shown to drive negative outcomes resulting from ADE in the case of Dengue and Zika viruses (101). Structural and functional characterization of cross-reactivity (102–105) has facilitated engineering efforts (31, 106, 107) to develop both broad-spectrum and highly-specific mAbs toward both lineage-agnostic treatment and lineage-specific diagnosis and treatment (108, 109).

As an example of such antibody repurposing against homologous protein epitopes, the wealth of existing tools available for these efforts, and also the limitations to their success, consider the identification of antibodies isolated from convalescent SARS patients that cross-recognized SARS-CoV-2 in the months following SARS-CoV-2’s emergence. Antibody CR3022 was isolated from a convalescent SARS-CoV patient in 2006 (110), and in the weeks following identification of SARS-CoV-2 researchers identified CR3022 as the first antiviral candidate against SARS-CoV-2 on the basis of RBD sequence conservation with SARS-CoV (111). Of note, other SARS-CoV neutralizing antibodies such as CR3014 and m396, which also shared target antigen sequence conservation, did not cross-neutralize SARS-CoV-2. Unfortunately, though, CR3022’s binding affinity for SARS-CoV-2 was significantly lower than its affinity for SARS-CoV, and CR3022 was found not to neutralize SARS-CoV-2. Still, due to CR3022’s rapid identification as a cross-binder significant engineering effort was pursued in hopes of obtaining a CR3022-derived mAb with enhanced therapeutic potential.

The structures of CR3022 bound to SARS-CoV and SARS-CoV-2 were rapidly solved (112), and experimental work determined that residue P384 drove the majority of the difference in CR3022 binding affinity between SARS-CoV and SARS-CoV-2, with the P384A mutation on SARS-CoV-2 restoring CR3022 binding affinity to that of its activity against SARS-CoV (113). These insights have since enabled engineering efforts to improve the therapeutic potential of CR3022 *via* molecular dynamics-based affinity maturation (114–116), directed evolution (116), framework engineering (117), and Fc engineering (118), demonstrating the widespread accessibility of tools for rapid repurposing and engineering of antibodies targeting homologous protein epitopes. Indeed, the directed evolution approach obtained a CR3022 derivative, eCR3022, with 1000-fold increased binding affinity for SARS-CoV-2 which resulted in restoration of neutralization (116). Despite this, structural analyses of SARS-CoV-2 spike dynamics and the CR3022 epitope showed that CR3022 could only bind to specific spike conformational states in which at least two RBDs were up (112), potentially limiting its neutralization potency independent of binding. This exemplifies the importance of understanding antigen dynamics when identifying candidate epitopes with high potential for translation to the clinic. Had early repurposing work instead identified an antibody against less complex spike epitopes exposed for all spike conformational states, it is plausible that the path to translation may have been quickly realized without the need for significant engineering. We next describe EPI analysis of a cross-neutralizing anti-Henipavirus antibody to demonstrate repurposing potential using accessible methods in the absence of complex epitope dynamics.

#### 2.1.2 Structure-Guided EPI Investigation of a Cross-Reactive Nipah and Hendra Virus Ab

We next examine a case of a cross-neutralizing antibody targeting a relatively simple protein epitope, highlighting how in the absence of epitope complexity existing methods enable cheap and accessible characterization of homologous epitope-paratope interactions despite the absence of a solved crystal structure. Nipah virus (NiV) is a zoonotic RNA virus of the Paramyxoviradae family (119), which includes measles and mumps. Following an incubation period of 5 to 14 days, NiV presents with fever, headache, and confusion, and then may progress to acute respiratory distress and encephalitis (120). Mortality rates range from 40-90%, making Nipah one of the deadliest viruses identified (121). While no vaccines or NiV specific therapeutics exist (122), antibody m102.4 which was isolated from a convalescent individual infected with the related Hendra virus (HeV) is known to cross-neutralize NiV. The epitope on HeV GP targeted by m102.3 is monomeric and aglycosylated within the antibody footprint, featuring a hydrophilic rim and a hydrophobic pocket in which the m102.3 CDR H3 is inserted. Further, 12 of 15 direct epitope-paratope interactions occur between the CDR H3 and the epitope, with 3 interactions occurring between the epitope and the LC. Additionally, the heavy chain that dominates the interaction is 100% conserved between m102.3 and m102.4. These features of the epitope and paratope make m102.3/4 a desirable candidate for repurposing and engineering, as no additional insight into the henipaviral GP biological context (e.g., dynamics, site-specific glycan identity, oligomerization, complex functional sites/domains) is required, and antibody engineering can be largely restricted to the CDR H3, framework region, and constant regions. While a structural model of the m102.3-HeV complex exists, there was no existing structural information on the cross-neutralization of NiV that provided a detailed structural representation of the m102.4-NiV antibody interaction.

We therefore leveraged a set of computational methods to model the NiV glycoprotein epitope bound by m102.4, and then select likely epitope and paratope hotspots for subsequent experimental investigation (92). These computational tools included homology modeling *via* ABodyBuilder (123), surface accessibility analysis, and significant interaction networks (91). We subsequently performed alanine scanning mutagenesis on the 19 selected paratope sites and 28 selected epitope sites, testing each mutant for protein expression, stability, and effect on the binding interaction. The data from the alanine scan was subsequently used to identify likely binding hotspots on the epitope and paratope, which were then incorporated to build a model of the interaction *via* docking (124) constrained by the likely EPI contact sites. The top model indicated high homology to the m102.4-HeV interaction, albeit with a slight rotation of the antibody in the binding pocket. Epitope features known to be important to the m102.3-HeV interaction from the existing structural model including interactions with the hydrophobic binding pocket and hydrophilic ring were largely conserved. This exercise demonstrates how available webtools and an accessible and cheap experimental workflow can be employed to map cross-reactive EPIs in the absence of significant epitope complexity and with the biological context of a related interaction.

#### 2.1.3 Glycans as Epitope Constituents Driving Epitope Complexity

In contrast to viral protein epitopes, repurposing of antiviral agents against viral glycoproteins whose surface proteins are heavily glycosylated has been less successful due to the biochemical complexity of EPIs comprising of or adjacent to N-glycans. Attempts using antibodies have largely been restricted to antibodies targeting protein surfaces on the target glycoproteins that are not shielded by glycans, prompting efforts to target emerging glycoproteins to often begin with surveys of non-shielded epitopes (28) rather than considering the full range of available neutralizing epitopes on the target glycoprotein. To our knowledge, there have no been successful rational antibody engineering campaigns against epitopes comprising N-glycans, in which investigators rationally selected paratope mutations to enhance the template antibody’s function based on an EPI model that suggested the selected mutations would result in more favorable paratope-glycan interactions. While this is likely due to the lack of computational tools to analyze and optimize EPIs involving N-glycans, we note that the intended outcome has been incidentally achieved demonstrating feasibility. Recently, anti-gp120 antibody VRC01 was engineered for 10-fold enhanced potency (derivate termed VRC01.23LS), and a structural model of the complex indicated that the enhanced breadth was likely driven in part *via* new hydrogen bonds between the paratope residue R66 and the N276 glycan (125). Indeed, rational engineering of antibodies interacting with N-glycans is complex as one must balance any mutation intended to enhance glycan-affinity with the potential for counter-productive disruption of the affinity for the nearby protein surface. Further, the dual glycan-protein recognition may focus antibodies toward specificity rather than desirable broad spectrum activity, further limiting anti-glycan antibody engineering and repurposing efforts.

Efforts to target glycans on viral surface glycoproteins have largely focused on the evaluating the use of lectins to specifically bind to the distinct viral surface glycans and thereby neutralize the virus. Lectins are carbohydrate-binding proteins found throughout the natural world, and in particular lectins derived from Algae including griffithsin and cynovirin-N recognize high-mannose N-glycans that are expressed on many viral glycoproteins such as HIV gp120, hepatitis C virus E1/E2, Ebola GP1,2, and SARS-CoV spike, resulting in potent neutralization of these viruses (126–131). Further, griffithsin recently completed a phase 1 clinical trial, demonstrating a strong safety profile as a topical prophylactic against HIV and other viruses including herpes simplex virus type-2 (132). While certain lectins explored for therapeutic use are mitogenic, recent work has shown that lectins are amenable to engineering toward enhanced safety profiles while retaining broad-spectrum and potent activity (133, 134). While these results are promising and we look forward to continued clinical development of lectins, this perspective and our work focuses on antibody EPIs. We therefore next describe a repurposing effort for the special case of antibody 2G12, which highlights the lack of publicly-available tools to analyze complex EPIs involving N-glycans, yet demonstrates that such efforts are tractable and enable repurposing of anti-glycan antibodies.

#### 2.1.4 Topological Model for Anti-Glycan Antibody 2G12 Cross-Reactivity

Epitopes presented at quaternary junctions and involving N-glycans are biochemically and structurally complex, and there is a lack existing analytical tools developed for their assessment. We therefore present a second modeling and repurposing effort for these more complex interactions using the antibody 2G12. 2G12 was discovered and rigorously characterized for its interaction with HIV gp120 (66, 135–137) and more recently 2G12 was observed to bind to Flu H3 HA (138) and SARS-CoV-2 spike (77). Toward exploring the breadth of 2G12 anti-Flu activity, we characterized a variety of 2G12-Flu interactions for H1N1 and H3N2 viruses. After observing strong neutralization or functional inhibition for a variety of Flu lineages, we became interested in identifying additional repurposing targets. However, to the best of our knowledge no existing webserver, algorithm, or tool existed to predict whether a glycan-recognizing antibody could bind to a given glycoepitope on the basis of a protein structure alone.

We subsequently hypothesized that additional 2G12 repurposing targets could be identified based on topological descriptions of N-glycan sites. Specifically, by identifying proteins with multiple N-glycan sites arranged such that they might induce oligomanosylation at the sites and subsequently present high-mannose N-glycans in an arrangement complementary to the primary and secondary 2G12 glycan-binding surfaces. We developed an algorithm to score protein structures based on these two features, and found that the resulting score correlated well with the ranking of apparent binding strength across Flu and Yeast glycoproteins that bind 2G12. That is, our results suggested that the algorithm score could have predictive value for identifying glycoproteins that bind 2G12 and for ranking these hits for subsequent experimental validation.

We therefore applied the model to generate a list of potential repurposing targets for 2G12. This process illustrates how conventional docking and binding prediction tools based on protein-protein interactions can be expanded to consider more complex interactions involving N-glycans. Such tools may see increased utility as the field is just beginning to appreciate the breadth of anti-glycan antibodies across viruses (77) and as structure prediction tools such as Alpha Fold 2 (139) and RosettaFold (140) greatly expand the range of structural data on emerging viruses toward antibody repurposing.

### 2.2 Tracking Viral Evolution in a Complex Epitope Landscape

#### 2.2.1 Modeling SARS-CoV-2 Variant Escape From Antibodies

Epitope complexity further results from the secondary and tertiary structures that form and support epitope surfaces, wherein these higher-order structures drive allosteric and epistatic effects that can critically affect antibody-antigen interactions and result in antibody escape. While much of the EPI can be understood from examining individual epitope and paratope residues in direct contact with each other *via* conventional annotations of amino acid interactions, epitope residues not in direct contact with a given antibody and networks of functionally-linked epitope residues may also contribute substantially to the epitope surface properties *via* allostery and epistasis. For a guiding example of allostery on the SARS-CoV-2 RBD, consider the recently characterized E406W mutation that resulted in subtle RBD structural changes that prompted escape from three clinical Abs (Casirivamab, Imdevimab, Cilgavimab) despite site 406 residing outside of the epitopes of all three Abs. While E406W is likely to be one of the more significant allosteric escape mutations on SARS-CoV-2 RBD, similar mechanisms may play a more local role for many escape mutations, especially when occurring in combination with other mutations as observed for the Omicron BA.1 and BA.2 subvariants. For an example of epistatic escape effects—or the synergistic effect resulting from combinations of mutations—consider the escape of BA.1 from antibody ADG20 (Adintrevimab). It was predicted that broad-spectrum and potent anti-SARS-CoV-2 antibody ADG20 would retain activity against the BA.1 Omicron sub-variant based on an analysis that ADG20 targets a highly sequence-conserved epitope surface and that the individual Omicron BA.1 mutations are not *individually* associated with escape *in vitro* (28, 141). However, ADG20’s potency against Omicron BA.1 was substantially reduced as compared to the D614G SARS-CoV-2 strain (142), highlighting the clear clinical relevance of epistasis as an epitope complexity factor. The mutation of critical epitope-adjacent sites and combinations of epitope mutations can therefore result in escape from antibodies *via* indirect effects that cannot be understood through conventional examination of individual direct EPI interactions.

We have modeled such indirect epitope effects using amino acid interface (AAI) network analysis (91), which quantitates the degree of structural and chemical connectivity between interface residues and the surrounding antigen structural context. On the other hand, similar information has also been generated experimentally through deep mutational scanning (DMS) techniques (11, 37, 39), in which all single mutations on a wild-type antigen (in this case the SARS-CoV-2 RBD) are mutated in parallel *via* yeast display and assayed for their effects on binding to a given antibody. Such assays are exceedingly useful for describing point mutation escape profiles for a given antibody or sera sample, but also limited in their ability to predict escape conferred by certain forms of epitope complexity, such as allosteric effects across tertiary or quaternary antigen structure as well as effects due to modulating antigen dynamics.

While AAI network analysis can identify tertiary and quaternary network effects, it is also limited in modeling of epitope complexity resulting from dynamic effects, unless applied over the course of a representative molecular dynamics simulation (MD) trajectory. To the best of our knowledge such network+MD analyses have yet to be performed, though are likely to be a fruitful research avenue. A contemporary example of epitope dynamics driving such complexity wherein current tools are limited in their application are the SARS-CoV-2 Omicron sub-variant mutations S371L and S371F mutations. S371L/F were recently observed to result in substantial escape from all four classes of anti-RBD antibodies in pseudoviral experiments (48, 49). Yet, neither DMS nor AAI measured a significant escape signal for class 1 and 2 RBD antibodies for any mutation at residue S371. This surprising result led us to suspect that the S371L/F mutations mediate their effect through a feature of epitope complexity that has not yet been described for SARS-CoV-2 spike. We hypothesized that epitope complexity at play is likely a combination of structural dynamics and N-glycan interactions, most likely mediated *via* a novel interaction between L/F371 and the N343 N-glycan (81). More specifically, we hypothesized the novel interaction may modulate the dynamic spike opening process *via* the previously described “glycan gate” function of the N343 glycan (83), in which the N343 glycan releases the adjacent RBD promoter from the three RBD-down (spike closed) conformation and then subsequently pushes the released RBD into the RBD-up state. Intriguingly, Sztain et al. further implicate residues D405 and R408 as participating in the glycan gate mechanism (83), which have been mutated alongside S371F on the BA.2 subvariant, suggesting potential epistasis or functional compensation. Whether such a dynamic effect explains the observed escape from S371L/F mutations remains to be experimentally validated, and we hope that the exact mechanism is elucidated soon.

Still, AAI and other escape-mapping techniques have proved valuable for rapidly assessing emerging SARS-CoV-2 variants and their escape mutations, in large part due to their modeling of indirect effects mediated by higher-order structure. We recently applied AAI to rapidly predict the impact of the Omicron BA.1 mutations on the set of clinically-authorized therapeutic antibodies in the days immediately following the first observation of BA.1 (52). We found that certain antibodies including ADG20 had multiple Omicron BA.1 mutations within the local epitope network, suggesting the potential for a large indirect perturbation of the epitope surface properties. This finding contrasted with experimental data on the impact of each BA.1 mutation in isolation on the binding and neutralization of ADG20, in which all individual BA.1 mutations were found to be tolerated by the antibody (28). Similar potential network-perturbations were observed by AAI for antibodies AZD8895 (Tixagevimab) and AZD1061 (Cilgavimab), which also retained activity against the BA.1 mutations in isolation, yet suffered significant drops in potency against the cumulative BA.1 mutations, which is most likely to be explained by indirect mechanisms. This case-study highlights the importance of considering epitope complexity resulting from indirect structural perturbations when rapidly evaluating the putative impact of an emerging variant bearing many mutations on the function of a therapeutic antibody.

**Figure 2:**
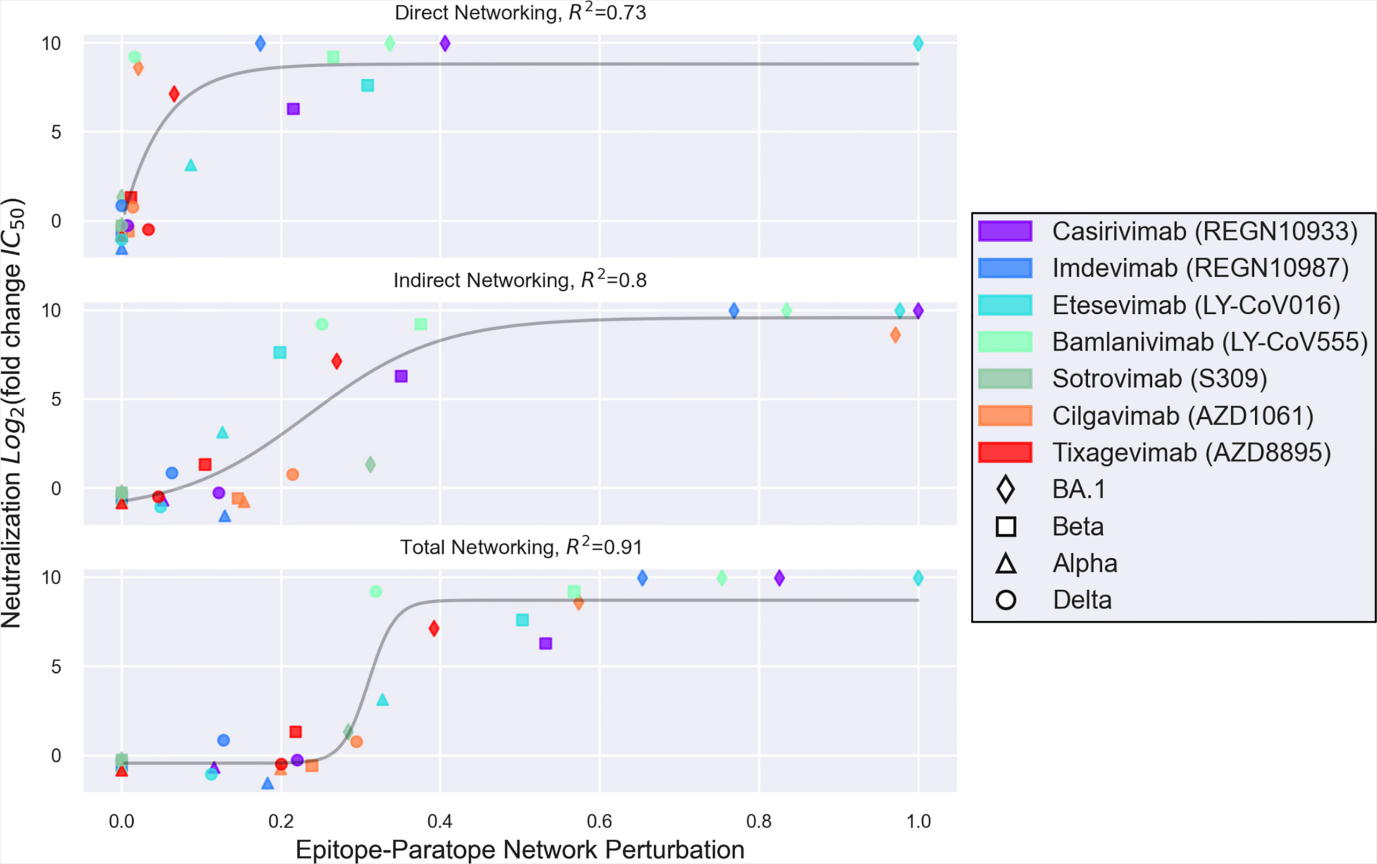
A set of 28 interactions between seven therapeutic antibodies and four SARS-CoV-2 variants are shown, wherein the y-axis depicts variant escape from antibody neutralization (Log_2_ fold reduction in IC_50_), and the x-axis depicts a networking perturbation score computed for the given variant-antibody epitope-paratope interaction. The epitope-paratope networking models were previously published by Miller et al. (52), and the pseudoviral escape measurements for each variant were published by Liu et al. (49). The top, middle, and bottom plots show direct, indirect, and total networking perturbations, respectively. While direct networking identifies most antibodies escaped by a given variant on the basis of direct epitope-paratope interactions, direct networking falls short of identifying a number of escape interactions which were also commonly missed by sequence- or point-mutation analyses and likely result from allosteric or epistatic interactions—key epitope complexity features. Meanwhile, indirect networking detects a perturbation for most of the escape interactions missed by direct networking, but still offers ambiguous readout for certain scores. Total networking, which combines both direct and indirect networking metrics, appears to provide the best model of variant escape from the set of RBD-directed therapeutic antibodies. Importantly, however, approaches such as AAI that model one aspect of epitope complexity (here *via* indirect networking) may still be limited by other epitope features driving epitope complexity such as glycosylation and protein dynamics.

Toward a more rigorous investigation of AAI for modeling the escape effect of SARS-CoV-2 VOC on therapeutic antibodies, we present next a comparison of 28 previously-published antibody-VOC escape interactions (**Figure 2**). This comparison was made possible in the months following the identification of Omicron BA.1 once experimental data on therapeutic antibody escape from Omicron BA.1 and several other VOC had been measured side-by-side (49). This comparison between AAI and experimentally-measured escape demonstrates how the *indirect* networking term of AAI substantially increases the modeling utility of the technique. Indeed, it is clear that models considering only *direct* interactions between the epitope and paratope are unable to identify all antibodies escaped by a given variant. Further, combining the direct and indirect features into a single Total Networking metric, which AAI uses for its primary prediction, produces a logistic relationship between modeled epitope perturbations and experimentally measured escape that appears to have predictive value. This comparison of direct, indirect, and total networking metrics broadly suggests that indirect effects—which result from various features of epitope complexity—are critical for modeling SARS-CoV-2 variant escape from RBD-directed antibodies.

In the months since the computational and experimental data in **Figure 2** on BA.1 were published, the BA.2 variant has outcompeted BA.1. Additionally, a number of new Omicron subvariants (BA.2.12.1, BA.4, BA.5) and recombinants (XD, XE) have emerged. While the AAI model from Miller et al., 2022 (52) as well as other escape calculators (60) are useful in the weeks following variant emergence to rapidly estimate therapeutic antibody escape—as will continue to occur—and further at later time points as tools for interrogating specific escape mechanisms, assessment of antibody escape for these new variants is not the goal of this review. Further, detailed experimental data on the escape of these sub-variants and recombinants has already begun to emerge, and we refer readers to these manuscripts (48, 143, 144). These recent data suggest that the BA.4 and BA.5 sub-variants are not only more immune-evasive than BA.1.1 and BA.2, but have also evolved to escape antibodies elicited by BA.1 infection (143, 144).

Importantly, computational techniques such as AAI as well as experimental scanning approaches include certain limitations. In the AAI analysis of the SARS-CoV-2 RBD, these limitations have been significant for two specific antibodies thus far. First, S309 (Sotrovimab) targets the N-glycan at residue N343 on RBD, yet as described earlier in this review epitopes including N-glycans are complex and poorly modeled by existing methods. Computational and experimental approaches for investigating S309 against variants are therefore limited unless they are validated in their ability to faithfully capture interactions with the S309 proteoglycan epitope, for example, *via* confirmation of biologically-correct glycosylation (e.g., correct species) on recombinant spike or RBD antigens. Second, in the AAI survey of variant-antibody interactions, we identified the BRII-196 (Amubarvimab) and Beta variant interaction to be incompletely modeled. Examining the direct and indirect features for this interaction, we found that the predicted escape results from a high direct networking perturbation. This is consistent with structural analysis of the interaction, which shows a hydrogen bond and hydrophobic interactions between paratope residues Y33, Y52 and epitope residue K417, suggesting that the K417N mutation on the Beta variant is likely to directly perturb the interaction. Yet, a structure between BRII-196 and the Beta variant has been solved, and indicates that the mutated N417 residue forms a new hydrogen bond with paratope residue Y52 (145). This outlier therefore highlights the difficulty in predicting epitope network perturbations in which select antibodies are capable of accommodating certain mutations.

In summary, epitope analysis that considers network complexity resulting from higher-order structures enables better modeling of allosteric and epistatic effects and offers improvement over conventional EPI annotations that consider only direct epitope-paratope interactions. These features can be captured by approaches such as the AAI indirect networking metric. However, such approaches that consider these aspects of epitope complexity can still be limited as a result of other aspects of epitope complexity which they may not capture such as glycan-protein interactions (as in the case of S309) and antigen structural dynamics (as in the case of the range of the spike trimer conformational states).

#### 2.2.2 Epitope Complexity Resulting From Epistasis

Further, experimental approaches have recently begun to elucidate an additional layer of epitope complexity conferred by epistasis, or combinations of mutations that together confer synergistic or novel effects exceeding the sum of each mutation’s individual effect. Mechanisms of epistasis may be either direct in a physiochemical manner or indirect *via* modulation of fitness landscapes (146). As shown by the significant disagreement between predicted performance of ADG20 against the BA.1 Omicron sub-variant from examination of each BA.1 mutation individually as compared to the true performance when assayed against all BA.1 mutations together, epistatic effects are highly relevant to EPIs. Recently, Starr et al. repeated their RBD DMS workflow employing the backbones of a number of SARS-CoV-2 variants to describe how the effect of each point mutation varies depending on the mutational context of RBD (147). Further, statistical models have found success in predicting SARS-CoV-2 site mutability from sequence data (148), and these epistatic interactions likely constrain the evolutionary landscape to drive future SARS-CoV-2 evolution. While these epistatic relationships may be driven largely by selection for enhanced fitness, such as the mutational pair Q498R and N501Y which together enhance ACE-2 binding (149), such epistatic effects are still relevant to EPI analyses as these residues are involved in numerous antibody epitopes. Given the strong overlap between neutralizing epitopes and functional sites on viral surface proteins, it is likely generally important to consider how epistasis driving enhanced fitness may also lead to escape effects at epitopes overlapping these functional sites.

#### 2.2.3 Bonus Epitope Complexity: Epitope Evolution Resulting From Polyclonal Responses

An additional aspect of epitope complexity arises when attempting to characterize future evolutionary paths. As host antigenic pressure contributes and, in many cases, drives future mutations, it is important to consider a given epitope in the context of adjacent and overlapping epitopes that are also targeted by polyclonal responses against the antigen. We analyzed this additional aspect of epitope complexity to model potential viral evolution of SARS-CoV-2 RBD and NTD (51). Specifically, we considered the mutability of epitope sites and the relationships between epitopes as constituents of polyclonal sera responses. By integrating these features together, we identified SARS-CoV-2 putative antigenic drift sites (PADS) as those antigen sites that 1) are not structurally- or functionally-constrained from mutating, 2) result in large direct or indirect epitope perturbations upon mutation, and 3) are antigenically orthogonal with existing VOC mutations such that mutation is likely to result in a broadened polyclonal escape profile.

The PADS predictions, which were presented as a map of antigenic space, were found to align with experimentally-measured escape across nine VOC (150). The VOC with the largest escape at the time of the analysis featured mutations that knocked down orthogonal compartments of the polyclonal response, and in which these mutations occurred at sites that broadly perturbed many epitope surfaces. The latter observation was particularly interesting, as it suggested that convergently evolved escape mutations such as E484K and L452R tended to occur within regions of epitope overlap such that these single mutations perturbed a large number of antibody epitopes. Further, a number of the sites identified as mutable and strongly epitope-perturbing were identified on the highly-evasive Omicron variant despite not being observed on other VOC, including Q493 and Y505. Other Omicron mutated sites were also identified in our antigenic space framework as occupying highly unique positions in antigenic space, such as G496 and Q498. These sites did not cluster with other major antigenic sites, but rather between major antigenic clusters suggesting these sites play an epitope-support role across RBD epitopes, perhaps highlighting epistatic functions.

While a potential limitation of this approach to SARS-CoV-2 evolution prediction using complex epitope features is that the modeling is based on wild-type structural data, new VOC have typically emerged from older lineages rather than the currently dominant one. This pattern contrasts with the step-like evolution observed for the seasonal variation of Flu, including the addition of N-glycans that are typically added to HA every 5-7 years (151). Still, the root-emergence pattern for SARS-CoV-2 preserves the length of time during which the PADS analysis remains relevant for interpreting constellations of mutations on new VOC. Further, a primary hypothesis for the VOC emergence pattern is that these variants are evolving during chronic infection, wherein these chronic infections were seeded months ago by the locally-circulating lineage at the time (152–154). This leaves open the possibility that a new strain evolved in a current chronic Alpha, Beta, or Delta infection could eventually emerge and replace Omicron. The PADS predictions offer starting escape landscapes for each of these variants, and facilitate predictions of additional mutations that may accrue most synergistically during continued chronic evolution.

## 3 Discussion

Epitope surfaces have long been viewed and defined at the level of amino acid sequence in a protein based on the ‘footprint’ of the paratope of an antibody binding to the surface of an antigen. The footprint is typically defined structurally by solving the structure of the antibody bound to the antigen and/or biochemically by analyzing mutations in the epitope that dramatically impact binding to an antibody (referred to as hotspots in an epitope for antibody interactions). This definition of epitope surface at the level of protein sequence at interface of antibody-antigen interaction has shaped understanding of epitope similarity between antigens and antibody engineering for targeting or enhancing affinity to a given antigen.

However, as highlighted in this review epitopes on viral surface antigens have several layers of complexity in epitope-paratope interactions owing to 1) the quaternary assembly and higher-order structure of protein domains on the viral surface, 2) protein glycosylation including at clustered sites (that lead to predominance of non-self surface glycans such as high-mannose type structures), and 3) large conformational transitions of the surface proteins owing to their role in both receptor binding and membrane fusion during various stages of maturation in the viral infection cycle.

In this review, we discuss aspects of epitope complexity that must be considered when targeting certain desirable viral antigens and when modelling and predicting viral evolution. As an example, the cross-neutralizing antibody engineering for Nipah virus case study describes a path for rapid response in the event a Henipavirus (or other virus with well-characterized homologous EPIs) were to emerge as a pandemic threat. This path would involve 1) rapid experimental testing of all antibodies known to target related G proteins to identify template cross-neutralizers, 2) structure prediction of the novel G protein and the top binding template antibodies, 3) rational computational design to enhance affinity and specificity of the templates against the novel NiV-like virus.

Our review also points to the role of protein glycosylation in going beyond masking sequence epitopes to generating novel glycoepitopes that have been underappreciated in the context of defining epitope targets for neutralization by antibodies. This is evident from our approach to define a glycoepitope model for the 2G12 antibody that specifically recognizing an N-glycan cluster motif (without any contact with the amino acids on the protein) that can be applied to search other pathogens for similar motifs thereby providing potential repurposing targets for 2G12. A similar rapid repurposing effort might prove fruitful in the case of an emerging influenza A H3N2 lineage bearing a new N-glycan proximal to the HA receptor binding site.

Understanding epitope complexity, particularly in terms of epitope networks and polyclonal epitope space, is also imperative for selecting and engineering therapeutic antibodies. As observed across SARS-CoV-2 VOCs, antigenic pressure resulting in escape mutations appears to reflect overlap of immunodominant antibody epitopes, regardless of whether a given VOC has emerged as it navigated population-level immunity or within a chronic infection. This has resulted in two formats of escape VOC emerging in the pandemic thus far: 1) Beta/Gamma-like VOC with 2-3 mutations in orthogonal and immunodominant epitope regions such that they efficiently escape from a significant fraction of polyclonal antibodies, and 2) Omicron-like VOC which feature a greater number of mutations that result in both escape breadth (mutations across orthogonal epitopes) and depth (mutations accumulated within epitopes).

In both VOC cases, many therapeutic antibodies isolated from convalescent individuals that heavily weight conventional design objectives such as very high neutralization potency were easily escaped. Instead, antibodies such as S309 with lower potency, but targeting an epitope distinct from population-immunodominance profiles are observed to have preserved function. These findings and our modeling therefore suggest that therapeutic antibodies should be designed as cocktails targeting orthogonal epitopes with lesser public immunodominance toward long-lived clinical relevance. Epitopes under high antigenic pressure in the population resulting from natural infection and vaccination should be avoided, even if these antibodies target an epitope that is less common and highly-conserved such as the epitope targeted by ADG20 that was conserved across many CoVs, as adjacent/overlapping epitopes may still exert sufficient pressure to prompt mutations with a large cumulative indirect effect on the conserved epitope. This further highlights the value of rational antibody engineering, as products can be by design distinct from common natural antibodies.

In the context of the epitope complexity highlighted in this review, viewing epitope surface just as an amino acid footprint at the sequence level has led to many challenges that point to key gaps in this view. Such sequence-based analyses also tend to simplify epitope similarity on basis of sequence conservation ignoring nuanced impact of structural constraints on mutability imposed by higher-order structure, glycosylation, and conformational dynamics. For example (28, 141, 142), the case study of ADG20 discussed in the body of this review, wherein ADG20 was predicted to be effective against Omicron BA.1 on the basis of assaying individual mutations, highlights the concept that sequence conservation misses important nuances on mutational constraints. Further this example demonstrates how epistatic relationships between combinations of mutations are a highly relevant form of epitope complexity (52). With advances in technologies such as cryo-EM to capture viral surface proteins in the appropriate context of their quaternary assembly and accessibility of molecular dynamics simulations to capture antigen structural dynamics, we observe a positive trend toward increased appreciation of epitope complexity features. We believe that ongoing efforts to develop computational tools that incorporate epitope complexity will lead to better prediction of viral escape and facilitate designing potent antibodies that can achieve broad spectrum neutralization of diverse viral strains.

## Author Contributions

All authors conceived the work. NM and TC performed the analyses. NM and RR wrote the manuscript. All authors contributed to the article and approved the submitted version.

## Funding

NM was supported in part by T32 ES007020/ES/NIEHS NIH.

## Conflict of Interest

The authors declare that the research was conducted in the absence of any commercial or financial relationships that could be construed as a potential conflict of interest.

## Publisher’s Note

All claims expressed in this article are solely those of the authors and do not necessarily represent those of their affiliated organizations, or those of the publisher, the editors and the reviewers. Any product that may be evaluated in this article, or claim that may be made by its manufacturer, is not guaranteed or endorsed by the publisher.
